# FTO Regulates Apoptosis in CPB2-Treated IPEC-J2 Cells by Targeting Caspase 3 Apoptotic Protein

**DOI:** 10.3390/ani12131644

**Published:** 2022-06-26

**Authors:** Jiaojiao Yang, Juanli Zhang, Xiaoli Gao, Ruirui Luo, Kaihui Xie, Wei Wang, Jie Li, Qiaoli Yang, Xiaoyu Huang, Zunqiang Yan, Pengfei Wang, Shuangbao Gun

**Affiliations:** 1 College of Animal Science and Technology, Gansu Agricultural University, Lanzhou 730070, China; yangjj@st.gsau.edu.cn (J.Y.); zhangjuanli456888@163.com (J.Z.); gxl18892@163.com (X.G.); luoruirui628@163.com (R.L.); xkh34567@163.com (K.X.); lijie5272@126.com (J.L.); yangql0112@163.com (Q.Y.); huanghxy100@163.com (X.H.); yanzunqiang@163.com (Z.Y.); wangpf815@163.com (P.W.); 2 College of Animal Science and Technology, Northwest A&F University, Xi’an 712100, China; wangw@st.gsau.edu.cn; 3 Gansu Research Center for Swine Production Engineering and Technology, Lanzhou 730070, China

**Keywords:** m6A, piglet diarrhea, IPEC-J2, CPB2 toxin, FTO

## Abstract

**Simple Summary:**

Fat mass and obesity associated protein (FTO) is a key demethylase in the process of bacterial diarrhea in piglets. However, the involvement of FTO in infectious diarrhea caused by *Clostridium perfringens type C* is not known. This study demonstrated that FTO plays an indispensable role in this type of diarrhea; and that the absence of FTO promotes apoptosis and inflammation in the intestinal porcine epithelial cell line-J2 (IPEC-J2). FTO targets Caspase 3 to affect the apoptosis of IPEC-J2 cells.

**Abstract:**

N6-methyladenosine (m6A) modification can accommodate mRNA processing, stability, and translation in mammals, and fat mass and obesity associated protein (FTO) is a vital demethylase in the m6A modification pathway. *Clostridium perfringens type C* (*C. perfringens type C*) causes diarrhea in piglets and has a serious impact on the pig industry. However, our understanding of the effect of m6A in the process of *C. perfringens type C* infectious piglet diarrhea (CPTCIPD) is limited. Here, an in vitro model of CPTCIPD was constructed by treating the intestinal porcine epithelial cell line-J2 (IPEC-J2) with *Clostridium perfringens* beta2 (CPB2) toxin, and the role of FTO was analyzed using quantitative real-time polymerase chain reaction, Western blotting, and flow cytometry. The results revealed that the overall RNA m6A contents at the tissue and cell levels were significantly up-regulated after *C. perfringens* infection (*p* < 0.05). FTO expression was significantly reduced in CPB2-treated IPEC-J2 cells. Functionally, FTO knockdown in the treated cells inhibited their proliferation and promoted apoptosis and the inflammation phenotype, whereas FTO overexpression had the opposite effects. Inhibiting FTO prolonged the half-life and up-regulated the expression of Caspase 3, leading to apoptosis. Therefore, this work explored the regulation of FTO in IPEC-J2 cells after CPB2 treatment and enhanced our understanding of the effect of the m6A modification in CPTCIPD.

## 1. Introduction

Piglet death caused by diarrhea is a serious problem in the pig industry. The morbidity and mortality of *C. perfringens type C* infectious piglet diarrhea (CPTCIPD) is more than 70%, which critically affects the economic benefits of pig farming [1]. Newborn piglets and suckling piglets are more likely to be infected with *C. perfringens type C* than adult pigs [2,3,4]. *Clostridium perfringens* beta2 (CPB2) is a major toxin produced by *C. perfringens type C* and is related to necrotizing enteritis and enterotoxemia in domestic animals, including pigs [5], chickens [6], cattle and buffalo [7,8], and horses [9]. Treatment of the intestinal porcine epithelial cell line-J2 (IPEC-J2) with CPB2 caused apoptosis and increased levels of inflammatory markers, and damaged intestinal barrier function [10,11]. Here, we constructed an in vitro cellular model via CPB2 treatment of IPEC-J2 cells to explore the regulation of CPTCIPD.

Recently, N6-methyladenosine (m6A) has been detected in mRNA, opening new avenues for the study of post-transcriptional regulation [12]. As the richest and most conserved reversible post-transcriptional modification of mRNA, approximately 0.1 to 0.4% of mRNAs are modified by m6A in mammals [13]. The modification process is dynamic and reversible, being catalyzed by methyltransferase and removed by demethylase enzymes. Methyltransferase is a complex enzyme, whose core is composed of methyltransferase-like 3 (METTL3) and METL14, which regulates m6A modification [14]. Demethylases, such as AlkB homolog 5 (ALKBH5) and fat mass and obesity related (FTO), can remove methyl groups from adenosine. Additionally, the methylated reading proteins can recognize the RNA modified by m6A and combine with it to exert a regulatory function [15].

FTO, encoded by the FTO gene, is expressed widely in animal tissues. Experiments in vitro have demonstrated that demethylase FTO exhibits a highly effective oxidative demethylation activity on m6A residues in RNA [16]. FTO regulates various pathological activities, such as obesity [17], diabetes [18], and cancer [19,20,21] via the demethylation of its target mRNA. Tang et al. [22] found that FTO was overexpressed in pancreatic cancer cells, and that FTO knockdown promoted apoptosis in the cells. FTO has also been found to promote the progression of gastric cancer [23,24,25]. Research by Zhang et al. showed that the glycogen synthase kinase 3 beta (GSK3β)/FTO/myeloid zinc finger 1 (MZF1)/c-MYC axis suppressed the development of colorectal carcinoma (CRC) [26]. Methylation modification is related to animal infectious intestinal diarrhea [27]. In a study of inflammatory bowel disease (IBD), m6A-modified mRNA was suggested to be involved in IBD pathogenesis [28]. During the infection of Enterotoxigenic Escherichia coli (ETEC), forkhead box O6 (FOXO6) interacts with the m6A methylase METTL3 to promote the transcription of GPR161 (encoding G protein-coupled receptor 161), which in turn regulates the expression of intestinal β-defensin [29]. In addition, during porcine epidemic diarrhea virus (PEDV) infection, m6A modification of the host RNA participates in the regulation of FTO gene expression and PEDV replication [30]. These studies indicate that m6A methylation may be important for the regulation of host defense against microbial infection, causing diarrhea. However, whether CPTCIPD is regulated by m6A methylation and its mechanism remain unclear. Therefore, this study started with the m6A demethylase FTO, by exploring the effect of FTO on CPB2-treated intestinal cells for further investigation of the involvement and role of m6A methylation in piglet diarrhea.

## 2. Materials and Methods

### 2.1. Statement of Ethics

The sample collection and experimental protocols were approved by the ethics committee of Gansu Agricultural University (under permit No. 2006-398).

### 2.2. Sample Collection

In this study, 30 healthy seven-day-old piglets (Yorkshire sow × Landrace boar) not infected with diarrhea-causing bacteria (pathogenic *E. coli*, *Salmonella*, and *C. perfringens*) were used, with five of the animals being randomly chosen as the control group (IC) to feed 1 mL of sterile culture solution daily, and the remaining 25 piglets being given 1 mL of *C. perfringens* type C culture medium (1 × 10^9^ CFU/mL) daily for five days [31]. Finally, the 5 piglets with the highest scores were selected as the susceptibility (IS) group and the 5 with the lowest scores were selected as the resistance (IR) group, according to the diarrhea score [31]. Fifteen piglets from the IC, IS, and IR groups were euthanized, and the jejunum and ileum tissues were rinsed with sterile PBS buffer (pH = 7.4), then quickly frozen in liquid nitrogen and stored at −80 °C until RNA extraction.

### 2.3. Preparation of the CPB2 Toxin Protein

The CPB2 toxin protein was extracted and purified using the protocol of Gao and Luo [10,11]. Briefly, the pET-28a-CPB2 plasmid carrying the CPB2 gene was constructed and transformed into BL21 competent cells. High-Affinity Ni-Charged Resin FF (GenScript, Piscataway, NJ, USA) was used to purify the expressed CPB2 protein, and the purity and expression of the CPB2 protein was assessed on 12% SDS-PAGE. Finally, endotoxins were removed using a ToxOut™ Rapid Endotoxin Removal Kit (AmyJet Scientific, Wuhan, China) following the provided protocol.

### 2.4. Cell Culture and Treatment

IPEC-J2 cells were bought from BNFUTURE (Beijing, China), and cultured in Dulbecco’s modified Eagle medium (DMEM)/F12 containing streptomycin (100 μg/mL), penicillin (100 U/mL), and 10% fetal bovine serum. The cells were grown at 37 °C with 5% CO_2_. IPEC-J2 cells (1 × 10^5^ cell/mL) were seeded in 6-well plates (Corning Inc., Corning, NY, USA), grown for 24 h, and then treated for 24 h with CPB2 toxin at 0, 15, 20, or 25 μg/mL. Cells without CPB2 toxin stimulation were used as the control group. There were three replicate wells in both the treatment group and the control group.

### 2.5. Cell Transfection

Vector pcDNA3.1 was obtained from Thermo Fisher Scientific (Shanghai, China). The FTO cDNA was synthesized and cloned into vector pcDNA3.1 via its Nhel and XhoI restriction sites (pcDNA3.1-FTO) for overexpression. GENEPHARMA (Shanghai, China) synthesized small interfering RNAs (siRNAs) targeting FTO (si-FTO) and a negative control siRNA (si-NC). Transfection was performed with Lipofectamine 2000 (Invitrogen, Waltham, MA, USA).

### 2.6. Total m6A Measurement

The TRIzol™ reagent (TransGen Biotech, Beijing, China) was used for the extraction of total RNA from the ileal tissue and the IPEC-J2 cells (1 × 10^7^). The total m6A content was determined using an EpiQuik m6A RNA Methylation Quantification Kit (Colorimetric) (AmyJet Scientific, Wuhan, China) according to the manufacturer’s instructions.

### 2.7. Determination of Cell Morphology and Viability

IPEC-J2 cells were plated in 96-well culture plate wells at densities of 5 × 10^3^/well, with three replicates for each group. Ten microliters of Cell Counting Kit-8 (CCK-8; Beyotime, Shanghai, China) solution were then placed in each well. After 2.5 h of incubation, the absorbance of the wells was recorded at 450 nm to calculate the cell viability. The cell morphology was examined under inverted microscopy (Olympus IX71, Tokyo, Japan).

### 2.8. Quantitative Real-Time Reverse Transcription Polymerase Chain Reaction (qRT-PCR) Analysis

Total RNA was extracted from IPEC-J2 cells (1 × 10^7^) or pig jejunal and ileal tissue, as described above. The cDNA was then produced from the total RNA using a reverse transcriptase kit (Accurate Biotechnology, Hunan, Changsha, China), and was used for qRT-PCR amplification using SYBR Green Realtime PCR Master Mix (Accurate Biotechnology, Hunan, Changsha, China) on a LightCycler 480 instrument (Roche Applied Science, Mannheim, Germany). GAPDH was used as an internal reference. The primer sequences are shown in Supplementary Table S1. The relative expression level was determined using the 2^−∆∆Ct^ method [32].

### 2.9. RNA Stability

After transfection of the IPEC-J2 cells with si-NC and si-FTO for 24 h, the culture medium with replaced with medium containing actinomycin D (5 μg/mL), and the culture was continued for different time periods (0, 3, or 6 h). RNA was extracted as described above. The TRIzol™ reagent (Invitrogen) was used to extract the total RNA, and Caspase 3 mRNA was measured using qRT-PCR.

### 2.10. Western Blotting

Total proteins of cells or of intestinal tissue were extracted in 0.1 mL of RIPA buffer containing 10 μL of PMSF (Servicebio, Wuhan, China). SDS-PAGE (12%) was used to separate the proteins in the cell or intestinal tissue lysates, which were then transferred onto PVDF membranes (Beyotime, Shanghai, China). The membranes were blocked using 5% skim milk and were incubated with primary antibodies overnight at 4 °C. The specific antibodies employed were: Anti-FTO (bs-7056R, Bioss Inc., Woburn, MA, USA; 1:1000), Anti-Caspase 3 (bs-0081R, Bioss; 1:500) and anti-beta-actin (bs-0061R, Bioss; 1:2000). After incubation with a secondary antibody (GB23303, Servicebio; 1:5000) labeled with horseradish peroxidase, the bands were visualized using a chemiluminescence (ECL) detection system (Bio-Rad, Hercules, CA, USA).

### 2.11. Flow Cytometry

Apoptosis was measured using flow cytometry. Briefly, IPEC-J2 cells (1 × 10^5^ cells/mL) were plated in six-well plates and grown overnight at 37 °C. The cells were then transfected with pcDNA3.1-FTO or si-FTO for 48 h, followed by treatment, and then treated with CPB2 toxin for 24 h, with three replicates per group. After washing with cold PBS, the cells were resuspended in binding buffer (195 μL) and then reacted with Annexin V-FITC (5 μL) and propidium iodide (PI; 10 μL). Cell apoptosis was then detected using a FACSCalibur flow cytometer (BD Biosciences, San Jose, CA, USA).

### 2.12. Statistical Analysis

Data were analyzed using GraphPad Prism 6.0 software (GraphPad Inc., La Jolla, CA, USA) and SPSS v.20 (IBM Corp, Armonk, NY, USA) software. Data are presented as mean ± the standard deviation (SD) and were analyzed using Student’s *t*-test (two-tailed test), or Duncan’s test in one-way (ANOVA) was used for significance analysis. *p*-values < 0.05 were considered statistically significant.

## 3. Results

### 3.1. Total m6A Contents Are Upregulated in Ileal Tissue and IPEC-J2 Cells

**Figure 1:**
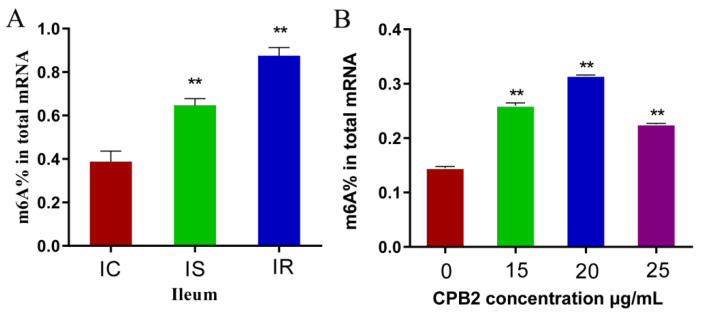
Total m6A contents of the ileum (**A**) and IPEC-J2 cells (**B**). (** *p* < 0.01).

The effect of m6A modification of RNA in piglet intestinal tracts of *C. perfringens*-infected piglets was first assessed by measuring the total methylation levels in the ileum of the IC, IS, and IR groups. The total m6A content of IS and IR groups was significantly higher than the IC group (Figure 1A). The overall methylation level in the CPB2-treated and control group IPEC-J2 cells was determined. This showed that the m6A content of the toxin-treated cells was significantly elevated (Figure 1B). These results, combined with the overall methylation levels in IPEC-J2 cells incubated with different CPB2 concentrations, allowed us to choose a concentration of CPB2 toxin of 20 μg/mL for subsequent experiments. In summary, the findings indicated that the overall levels of m6A methylation were related to CPTCIPD.

### 3.2. CPB2 Treatment Downregulated FTO in IPEC-J2 Cells

**Figure 2:**
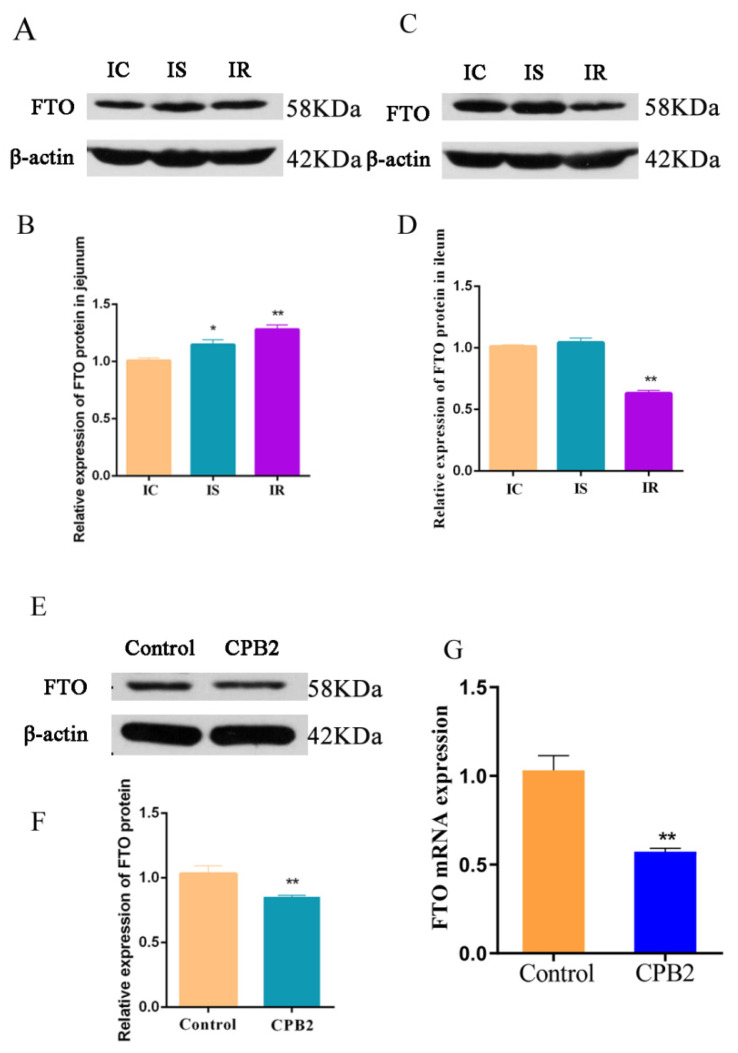
FTO expression in jejunal and ileal tissue, and IPEC-J2 cells. FTO protein levels shown via Western blotting in the jejunum (**A**,**B**), ileum (**C**,**D**), and cells (**E**,**F**). (**G**) FTO mRNA levels in CPB2-treated IPEC-J2 cells, measured via qRT-PCR. Cells were incubated with 20 μg/mL CPB2 toxin for 24 h; control cells received no treatment. * *p* < 0.05; ** *p* < 0.01. Original western blot figures in Figure S1.

Analysis of the FTO levels in jejunal and ileal tissue indicated that FTO was expressed to a significantly greater extent in the IS and IR groups in the jejunum, compared with the IC group (Figure 2A,B), and that FTO expression was reduced in the ileal IR group, in comparison with the IC group (Figure 2C,D). These results suggested that the FTO protein levels in different intestinal tissues varied. After CPB2 toxin treatment for 24 h, FTO protein (Figure 2E,F) and mRNA expression (Figure 2G) in the IPEC-J2 cells were significantly decreased. These findings suggested that in the IPEC-J2 cells, CPB2 toxin reduced FTO expression.

### 3.3. FTO Influenced the Viability of CPB2-Treated IPEC-J2 Cells

**Figure 3:**
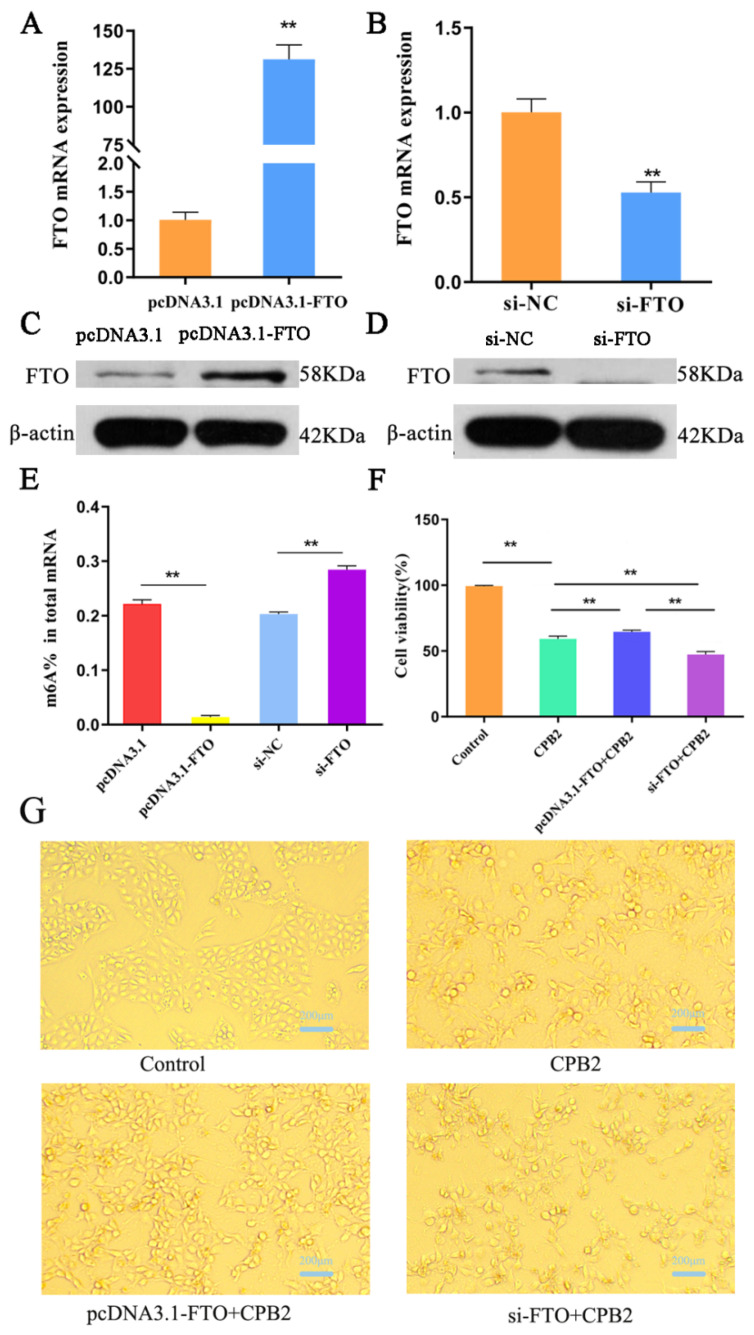
FTO influences on the viability of CPB2 toxin-treated IPEC-J2 cells. qRT-PCR (**A**,**B**) and Western blotting (**C**,**D**) confirmation of FTO knockdown and overexpression in transfected IPEC-J2 cells. The total m6A content in FTO-overexpressing or silenced IPEC-J2 cells (**E**). Effect of CPB2 on the viability of FTO overexpressing or silenced IPEC-J2 cells (**F**). The morphological changes in CPB2-treated FTO overexpressing or silenced IPEC-J2 cells. Scale bar = 200 μm (**G**). (** *p* < 0.01). Original western blot figures in Figure S1.

FTO was overexpressed or knocked down to study FTO’s role in IPEC-J2 cells. It was found that the levels of both FTO mRNA and protein were significantly reduced after FTO knockdown, while they increased significantly following FTO overexpression (Figure 3A–D). These results verified the successful overexpression and knockdown of FTO. FTO knockdown led to a marked increase in the overall m6A levels in comparison with the controls, while the m6A levels were markedly reduced after FTO overexpression (Figure 3E).

The cell viability assay showed that IPEC-J2 cell viability was significantly reduced to 59.2% after CPB2 toxin treatment; the cell viability in the pcDNA3.1-FTO + CPB2 group (64.56%) was significantly greater than in the CPB2 group; and the cell viability in the si-FTO + CPB2 group (47.26%) was significantly lower than that of CPB2 and pcDNA3.1-FTO + CPB2 groups (Figure 3F). Thus, FTO overexpression increased cell viability, whereas FTO knockdown decreased cell viability.

Morphologically, normal IPEC-J2 cells had a uniform morphology and good adherent growth. After CPB2 toxin treatment, the cells lost their adherence and the morphology was abnormal (such as shrinking and rounding). FTO knockdown intensified the changes in cell morphology, while overexpression reduced the phenomenon of cell rounding and shedding (Figure 3G).

### 3.4. The Effects of FTO on the Expression of Inflammatory Markers in CPB2-Treated IPEC-J2 Cells

**Figure 4:**
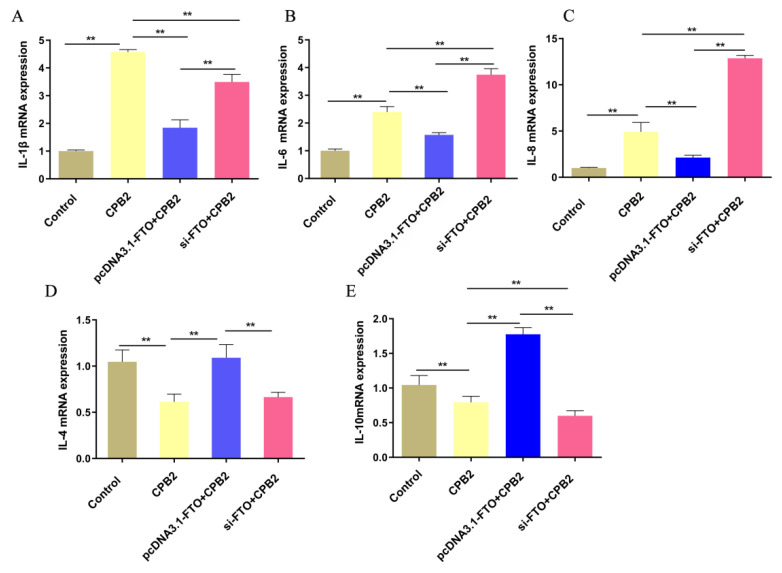
The effect of FTO overexpression or silencing on inflammatory factor expression. (**A**–**E**) *IL-1β*, *IL-6*, *IL-8*, *IL-4*, and *IL-10* mRNA levels in IPEC-J2 cells after treatment with 20 μg/mL CPB2 for 24 h. ** *p* < 0.01.

The mRNA levels of the inflammatory cytokines *IL-1β*, *IL-6*, *IL-8*, *IL-4*, and *IL-10* were determined via qRT-PCR. Figure 4A–C shows that CPB2 toxin treatment elevated the expression of *IL-8*, *IL-6*, and *IL-1β*. However, the mRNA levels of these cytokines were significantly reduced in the pcDNA3.1-FTO + CPB2 group, in comparison with the CPB2-treated cells, while those of *IL-6* and *IL-8* in the si-FTO + CPB2 group were significantly elevated. *IL-4* and *IL-10* expression were markedly higher in the pcDNA3.1-FTO + CPB2 group than in the CPB2 group, but significantly lower in the si-FTO + CPB2 group than in the pcDNA3.1-FTO + CPB2 group (Figure 4D,E). Overall, the overexpression of FTO lowered the levels of *IL-8* and *IL-6*, and promoted those of *IL-10*, while the knockdown of FTO showed the opposite results.

### 3.5. The Effects of FTO on CPB2-Induced Apoptosis in IPEC-J2 Cells

**Figure 5:**
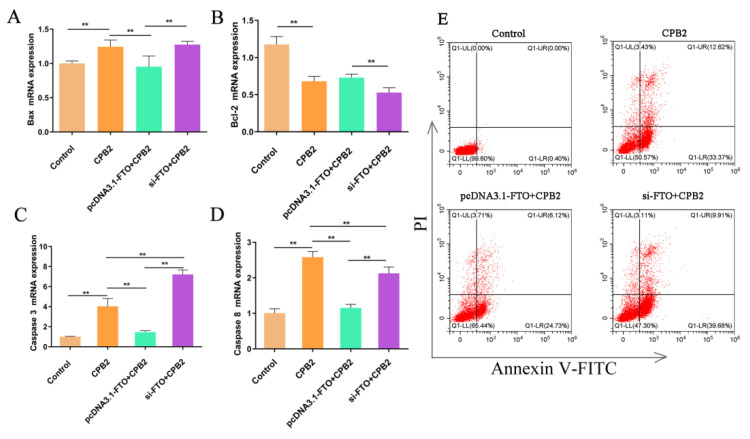
The effect of FTO on apoptosis in CPB2-treated IPEC-J2 cells. mRNA expression of *Bax* (**A**), *Bcl-2* (**B**), *caspase 3* (**C**), and *caspase 8* (**D**) in cells treated with 20 μg/mL for 24 h. Flow cytometry analysis of apoptosis in FTO-overexpressing or silenced cells treated with CPB2 (**E**). ** *p* < 0.01.

The influence of FTO on apoptosis in CPB2-treated IPEC-J2 cells was investigated in FTO-silenced or -overexpressing cells after treatment with CPB2 for 24 h. The mRNA expression of the apoptosis-related genes *Bax* (encoding BCL2 associated X, apoptosis regulator), *Bcl-2* (encoding BCL2 apoptosis regulator), *Caspase 3* (encoding Caspase 3), and *Caspase 8* (encoding Caspase 8) was measured (Figure 5A–D). It was found that the expression of the pro-apoptotic genes *Bax*, *Caspase 3*, and *Caspase 8* were significantly raised in the CPB2 group, while that of the anti-apoptotic *Bcl-2* was significantly decreased. *Bax*, *Caspase 3*, and *Caspase 8* in the pcDNA3.1-FTO + CPB2 group were significantly reduced in comparison with the CPB2 group, while the expression of these genes in FTO-silenced cells was significantly higher than in the pcDNA3.1-FTO + CPB2 group. The mRNA levels of *Bcl-2* in the si-FTO + CPB2 group were reduced compared with those in the pcDNA3.1-FTO + CPB2 group.

Cell apoptosis was also detected using flow cytometry (Figure 5E). There was almost no apoptosis in the control group, while the apoptosis rate reached 45.99% after CPB2 treatment. After overexpression of FTO, the apoptosis rate of the CPB2 toxin-treated cells decreased to 30.85%, while after the knockdown of FTO, the apoptosis rate of CPB2 toxin-treated cells increased to 49.59%. These results showed that the overexpression of FTO inhibited CPB2-induced IPEC-J2 cell apoptosis, while FTO knockdown promoted cell apoptosis.

### 3.6. FTO Targets Caspase 3 in IPEC-J2 Cells

**Figure 6:**
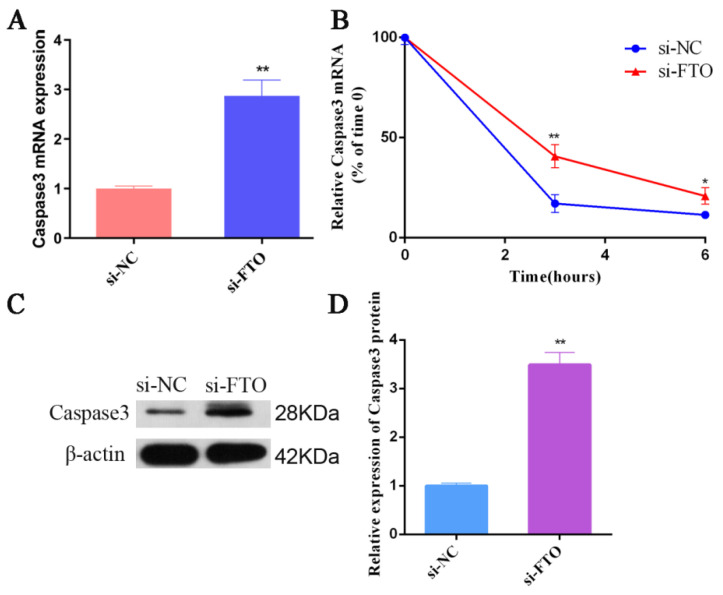
Inhibiting FTO promotes the expression of Caspase 3 in IPEC-J2 cells. (**A**) Upregulation of *Caspase 3* mRNA expression after FTO silencing. (**B**) RNA stability assay showing the half-life of *Caspase 3* mRNA after silencing of FTO. (**C**,**D**) Western blotting showing the Caspase 3 protein level after *FTO* silencing. * *p* < 0.05; ** *p* < 0.01. Original western blot figures in Figure S1.

Bioinformatics analysis using online tools (http://www.cuilab.cn/sramp, accessed on 8 September 2021) showed that Caspase 3 contained four GGACU motifs consistent with FTO binding. To verify whether FTO and Caspase 3 have a potential targeting regulatory relationship, we knocked down FTO in IPEC-J2 cells and detected *Caspase 3* mRNA expression. Significant elevations in Caspase 3 expression were observed in the FTO knockdown cells compared with the controls (Figure 6A). After treating cells with actinomycin D to block RNA synthesis, the data showed that reduced FTO expression increased the half-life (t1/2) of Caspase 3 significantly (Figure 6B). Consistent with the mRNA expression trend, the levels of Caspase 3 protein in the FTO-silenced cells were elevated in comparison with the controls (Figure 6C,D). These results indicated that FTO and Caspase 3 have a negative regulatory relationship, and knocking down FTO increased the half-life of Caspase 3, leading to increased Caspase 3 protein levels.

## 4. Discussion

The m6A modification of RNA is an important factor in epigenetic regulation, and has attracted increased research attention. In various pathophysiological processes, the m6A modification is believed to regulate RNA transcription and protein generation extensively [33,34]. Moreover, the m6A modification has a crucial impact on immune and inflammatory responses. For example, METTL3-mediated m6A modification plays a role in increasing the translation of some immunity-related transcripts, which accelerates dendritic cell (DC) activation and the DC-T cell response [35,36]. Previous studies have shown that piglets have varying degrees of diarrhea and intestinal inflammatory disease after infection with *C. perfringens type C* [31].However, there is little information on m6A methylation in CPTCIPD. Here, we detected the overall level of m6A methylation in the ileum tissue of piglets in the IC, IS, and IR groups, and the findings indicated significantly raised overall methylation levels in the IS and IR groups infected with *C. perfringens type C*. Abnormal methylation modification had close ties with the abnormal physiological state of the body. Wang compared the m6A content in cervical cancer tumors and their matched normal controls at the histological level; and found that m6A content in the tumor tissues was reduced and positively correlated with cancer progression [21]. In addition, Yang et al. investigated human lens epithelial cells (HLECs) after treatment with high glucose, observing higher m6A methylation in these cells [37]. Curcumin’s protective effect on liver injury induced by lipopolysaccharide and hepatic lipid metabolism disorders might be related to an increase in m6A methylation of RNA [38]. Meanwhile, levels of the demethylase FTO were elevated in the jejunal tissue of the IS and IR groups, and significantly decreased in the ileal tissue of the IR group. These findings indicate that m6A modification is associated with CPTCIPD.

The influence of m6A-mediated RNA methylation on the development of CPTCIPD was investigated by treating IPEC-J2 cells with the CPB2 toxin (20 μg/mL) to construct an in vitro model of diarrhea in piglets [10,39]. The overall m6A methylation content was found to be elevated, while the expression of FTO was significantly reduced in the treated cells. These results indicated the successful construction of the cellular model.

As the first identified methylation-related enzyme, FTO has vital functions in the m6A modification of mRNA [16,40], acting as an m6A regulator related to cancer cell apoptosis [41]. Using FB23-2 to inhibit FTO significantly down-regulated proliferation and promoted the apoptosis of human acute myeloid leukemia (AML) cells and primary embryonic AML cells [42]. After knockdown of FTO in bladder cancer cells, bladder cancer cells increased apoptosis and decreased cell proliferation and cell invasion [43]. In cocl2-induced human neuroglioma H4 cells, the knockdown of FTO facilitated cell apoptosis by activating caspase and arresting the cellular G1/S cycle [44].

Here, we observed that the knockdown of FTO resulted in the accumulation of the pro-apoptotic proteins *Caspase 3*, *Caspase 8*, and *Bax*, and reduced that of *Bcl-2* in CPB2-treated cells, while FTO overexpression produced the opposite effects. Meanwhile, FTO overexpression led to increased proliferation and reduced apoptosis in the CPB2-treated cells. Apoptosis is a strictly regulated form of programmed cell death and includes two pathways, namely, the intrinsic and extrinsic apoptotic pathways. Intrinsic apoptosis is controlled by proteins belonging to the Bcl-2 family and includes pro-apoptotic proteins such as Bax, Bad, Bak, Bid, Bim, Noxa, and Puma, as well as the anti-apoptotic proteins Bcl-2, Bcl-w, Bcl-xL, and Mel-1 [45,46]. These proteins are expressed in the mitochondria where they regulate membrane permeability, cytochrome C release, the activation of initiating caspases (such as Caspase 9), and the subsequent activation of effecting caspases (such as Caspase 3) [47,48]. Bcl-2 is associated with apoptosis and can be induced by lipid peroxidation, glucose deprivation, and growth factor deprivation [49]. Previous studies have shown that a high level of Bcl-2 expression can inhibit cell apoptosis [50]. Studies have shown that Bax can alter mitochondrial membrane permeability and activate nucleases and caspases, which leads to irreversible mitochondrial damage and accelerated programmed cell death [51,52]. Caspase 8 is involved in early apoptosis signal transduction, and is related to death receptors [53]. Once Caspase 8 is activated, it can immediately enable downstream effector caspases (Caspase 3 and Caspase 7) and facilitate cell death [54]. The poisonous terpenoid cantharidin induces apoptosis through Caspase 3 activation [55]. In summary, we speculate that the endogenous apoptotic pathway is involved in FTO-mediated CPB2-treated IPEC-J2 cell apoptosis.

FTO specifically binds to GGACU motifs, thereby removing the m6A modification [56]. In the present study, we identified GGACU motifs in Caspase3, suggesting that FTO might bind to Caspase3. To verify whether there is a targeting relationship between FTO and Caspase3, we silenced FTO in the cells. This resulted in increased *Caspase3* mRNA levels through the prolongation of the mRNA half-life. Therefore, we hypothesized that inhibition of the demethylase FTO enhances Caspase 3 expression and accelerates apoptosis after CPB2 treatment, although the specific regulatory mechanism of FTO on CPTCIPD needs to be further investigated in piglets in vivo.

FTO is associated with inflammation [57]. For example, FTO expression was negatively associated with *IL-6* expression in the adipose tissue of obese patients [58]. The m6A demethylase FTO regulates cardiomyocyte apoptosis and inflammation via YAP1 in ischemia–reperfusion injury [59]. Yu et al. found that alcohol increased PPAR-α m6A methylation via the FTO-mediated epigenetic modification of YTHDF2, ultimately leading to NLRP3 inflammasome activation and NF-κB-driven renal inflammation [60]. In this study, FTO overexpression was found to suppress the expression of *IL-8*, *IL-6,* and *IL-1β*, while promoting that of *IL-4* and *IL-10*, while FTO knockdown had the opposite effect, suggesting that FTO influenced inflammation in CPB2-treated cells. These findings may suggest novel ways of preventing and treating CPTCIPD.

## 5. Conclusions

In summary, CPTCIPD is closely related to m6A methylation modification. Inhibition of the demethylase FTO could promote both apoptosis induced by CPB2 treatment and inflammatory responses in intestinal epithelial cells. Moreover, the overexpression of FTO could reverse this phenomenon. The inhibition of FTO prolonged the half-life of *Caspase3*, thereby up-regulating the levels of Caspase 3 protein and promoting the apoptosis of IPEC-J2 cells. Hence, we hypothesize that FTO-mediated inflammation and apoptosis has an important role in the development of CPTCIPD.

## Data Availability

Data is contained within the article or Supplementary Material.

## Supplementary Materials

The following supporting information can be downloaded at: https://www.mdpi.com/article/10.3390/ani12131644/s1. Table S1: Information for primers used for qRT-PCR, Figure S1: Original western blot figures for Figure 2A,C,E, Figure 3C,D and Figure 6C.
