# Supplementary material for: FTO Regulates Apoptosis in CPB2-Treated IPEC-J2 Cells by Targeting Caspase 3 Apoptotic Protein
Source: Animals (Basel). 2022 Jun 26;12(13):1644. doi: 10.3390/ani12131644 (PMC9264887; doi:10.3390/ani12131644)
Supplement: Supplementary file 1 [file animals-12-01644-s001.zip › animals-1727930-Table S1.pdf]

Table S1 Information of primers used for qRT-PCR

Genes		Nucleotide Sequence (5'-3')	Product Length (bp)	Accession No
GAPDH	Forward	AGTATGATTCCACCCACGGC	139	NM_001206359.1
	Reverse	TACGTAGCACCAGCATCACC		
FTO	Forward	GCATGGCTGCTTATTTCGGG	154	NM_001112692.1
	Reverse	TGCATCAGAGCCCTTCACTG		
Caspase 3	Forward	CCGAAATGTTTGCTGACGGC	152	XM_013998624.2
	Reverse	CCGATCTCGAAGGAAGTCCA		
Caspase 8	Forward	CGGCTCTGAGCAAGACCTTTA	173	XM_021074714.1
	Reverse	GCCGTAGATGATGCCCTTGT		
Bax	Forward	GCTGACGGCAACTTCAACTG	202	XM_013998624.2
	Reverse	GCGTCCCAAAGTAGGAGAGG		
Bcl-2	Forward	GGTGAACTGGGGGAGGATTG	130	XM_021099602.1
	Reverse	GTGCCGGTTCAGGTACTCAG		
IL-1β	Forward	AAGCCGATGAAGAATCCCTC	146	XM_021085847.1
	Reverse	CACTCCATAGACTGCACGTTG		
IL-4	Forward	TTCGGCACATCTACAGACACC	101	NM_214123.1
	Reverse	TTCATGCACAGAACAGGTCA		
IL-6	Forward	TCTGGTGATGGCTACTGCCT	95	NM_214399.1
	Reverse	CCGGAGAGGTGAAGAGCATTT		
IL-8	Forward	CTGCAGCTCTCTGTGAGGCTGC	199	NM_213867.1
	Reverse	TCCTTGGGGTCCAGGCAGACC		
IL-10	Forward	GCCCTGTGAAAACAAGAGCAA	137	NM_214041.1
	Reverse	TCTTCATCGTCATGTAGGCTT		

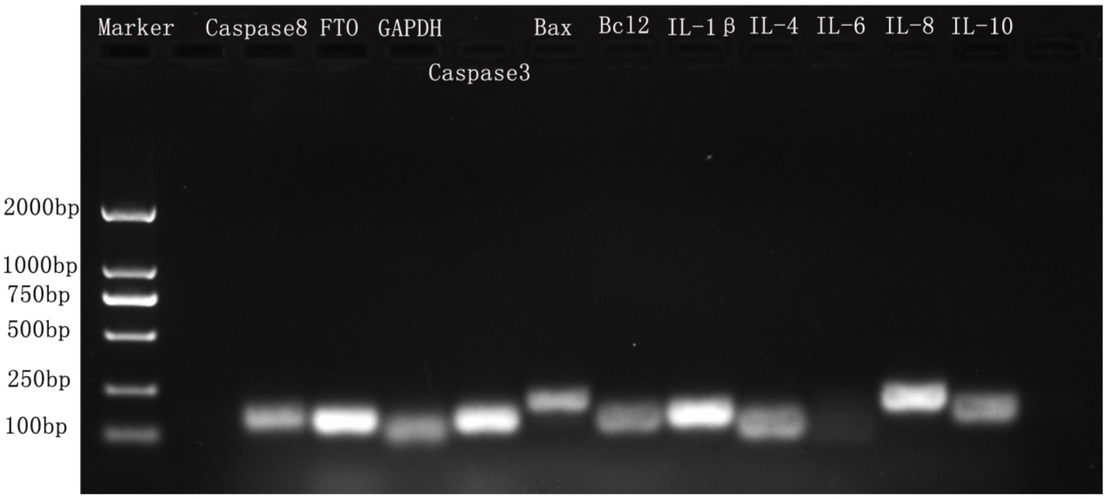

PCR amplification product of different primers
