# Supplementary figures and images for: FTO Regulates Apoptosis in CPB2-Treated IPEC-J2 Cells by Targeting Caspase 3 Apoptotic Protein
Source: Animals (Basel). 2022 Jun 26;12(13):1644. doi: 10.3390/ani12131644 (PMC9264887; doi:10.3390/ani12131644)

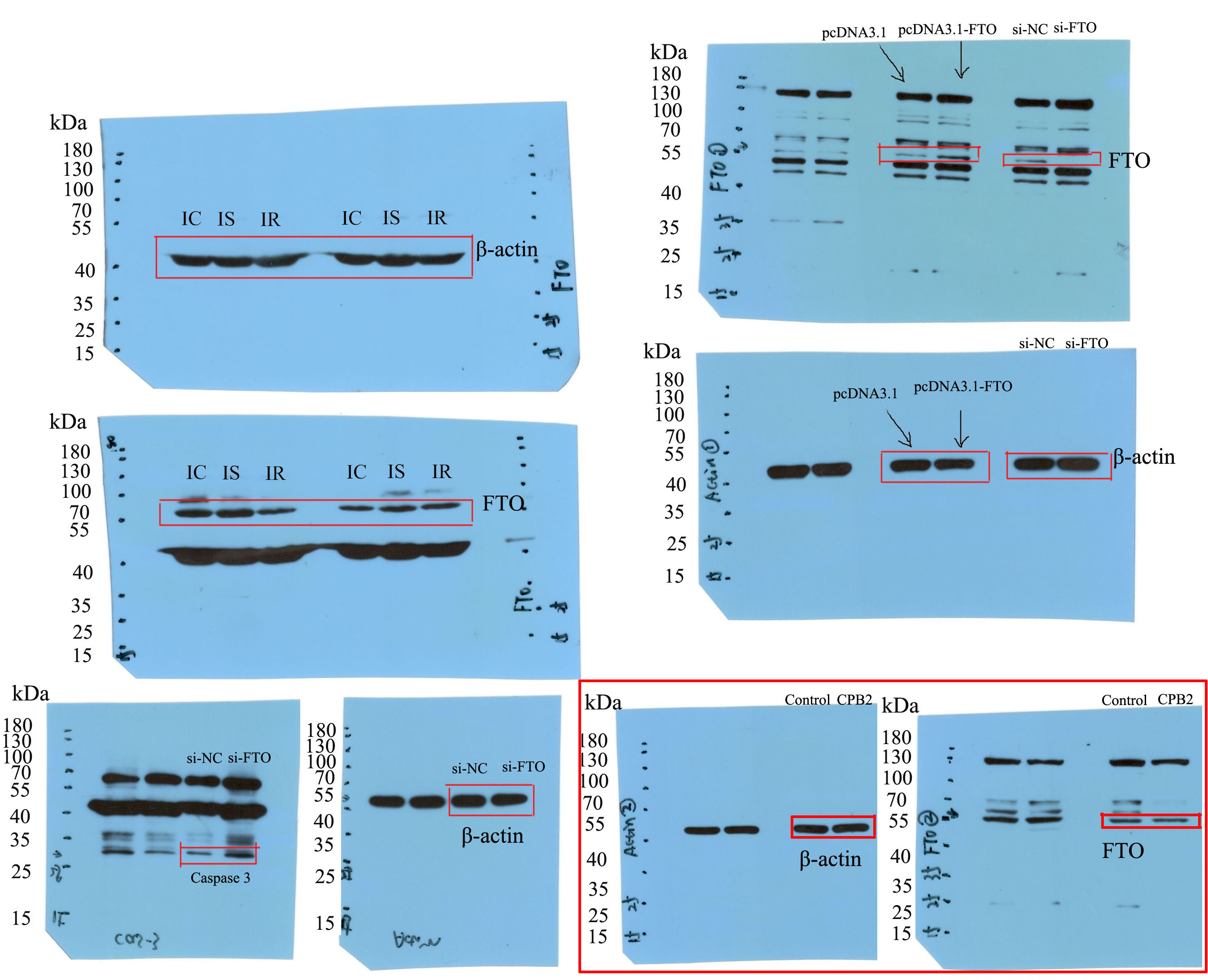

Supplement: Supplementary file 1 [file animals-12-01644-s001.zip › animals-1727930-Figure S1.jpg]
